# Preconception micronutrient supplementation positively affects child intellectual functioning at 6 y of age: A randomized controlled trial in Vietnam

**DOI:** 10.1093/ajcn/nqaa423

**Published:** 2021-03-01

**Authors:** Phuong H Nguyen, Melissa F Young, Lan Mai Tran, Long Quynh Khuong, Thai Hong Duong, Hoang Cong Nguyen, Truong Viet Truong, Ann M DiGirolamo, Reynaldo Martorell, Usha Ramakrishnan

**Affiliations:** International Food Policy Research Institute, Washington, DC, USA; Thai Nguyen University of Pharmacy and Medicine, Thai Nguyen, Vietnam; Emory University, Atlanta, GA, USA; Thai Nguyen National Hospital, Thai Nguyen, Vietnam; Hanoi University of Public Health, Hanoi, Vietnam; Thai Nguyen University of Pharmacy and Medicine, Thai Nguyen, Vietnam; Thai Nguyen National Hospital, Thai Nguyen, Vietnam; Thai Nguyen National Hospital, Thai Nguyen, Vietnam; Thai Nguyen University of Pharmacy and Medicine, Thai Nguyen, Vietnam; Georgia State University, Atlanta, GA, Georgia; Emory University, Atlanta, GA, USA; Emory University, Atlanta, GA, USA

**Keywords:** preconception, multiple micronutrients, child growth, child intellectual functioning, randomized controlled trial, Vietnam

## Abstract

**Background:**

Although there is growing evidence on the role of preconception nutrition for birth outcomes, very few studies have evaluated the long-term effects of nutrition interventions during the preconception period on offspring cognitive outcomes.

**Objective:**

We evaluate the impact of preconception weekly multiple micronutrients (MMs) or iron and folic acid (IFA) supplementation compared with folic acid (FA) alone on offspring intellectual functioning at age 6–7 y.

**Methods:**

We followed 1599 offspring born to women who participated in a double-blinded randomized controlled trial of preconception supplementation in Vietnam. Women received weekly supplements containing either 2800 μg FA only, 60 mg iron and 2800 μg FA, or MMs (15 micronutrients including IFA) from baseline until conception, followed by daily prenatal IFA supplements until delivery. We used the Wechsler Intelligence Scale for Children to measure full-scale IQ (FSIQ) and 4 related domains of intellectual functioning [Verbal Comprehension Index (VCI), Perceptual Reasoning Index (PRI), Working Memory Index (WMI), and Processing Speed Index (PSI) scores] at 6–7 y. Group comparisons were done using ANOVA tests for all children and the subgroup born to women who consumed the supplements ≥26 wk before conception (per-protocol analyses).

**Results:**

The final sample with data at 6–7 y (*n* = 1321) was similar for baseline maternal and offspring birth characteristics and age at follow-up by treatment group. Compared with the offspring in the FA group, those in the MM group had higher FSIQ (β = 1.7; 95% CI: 0.1, 3.3), WMI (β = 1.7; 95% CI: 0.2, 3.2), and PSI (β = 2.5; 95% CI: 0.9, 4.1). Similar findings were observed in the per-protocol analyses. There were no significant differences by treatment group for VCI and PRI.

**Conclusions:**

Preconception supplementation with MMs improved certain domains of intellectual functioning at age 6–7 y compared with FA. These findings suggest the potential for preconception micronutrient interventions to have long-term benefits for offspring cognition.

## Introduction

Poor growth and development during early childhood continue to be significant public health problems worldwide, particularly in low- and middle-income countries, where it is estimated that >30% of children younger than 5 y are stunted and 249 million children are not reaching their full developmental potential (1). Targeted nutrition interventions before and during the first 1000 d of life have the potential to improve child growth and development and subsequent intellectual functioning and human capital formation (2, 3).

Pregnancy and early childhood are periods of rapid growth and development in humans. Growth and development in motor, mental, and socioemotional domains continue at a rapid pace during the early years and are critical for shaping intelligence, personality, and social behavior, as well as for learning preparedness during the school years (4). The prenatal and early postnatal periods are characterized by increased nutrient requirements, susceptibility to illness, and vulnerability to inadequate care, but gaps remain in our understanding of the optimal timing of nutrition-specific interventions (5, 6).

Maternal nutritional status at conception can influence placental development, energy partitioning, and epigenetic remodeling of fetal genes (7). Several micronutrients that play an important role in brain development are often lacking in many diets (5). For example, iron is required by enzymes involved in many different brain functions, oxygen metabolism, cell division, myelination, and synaptic development (8, 9), all of which are important for cognitive development (10). Many other micronutrients and fatty acids likewise play critical roles in different early neurodevelopmental processes, including neuron proliferation, axon and dendrite growth, synapse formation, pruning, and function, myelination, and neuron apoptosis (11, 12). Provision of micronutrient supplements during the preconception period therefore may improve nutrient stores at the time of conception and during pregnancy, resulting in improved maternal and child health outcomes. Animal studies support the importance of maternal nutrition during the periconceptional period for offspring health and development (7, 13), but evidence from human intervention trials is sparse. Although there is growing evidence on the role of preconception nutrition for improved birth outcomes (7) and the demonstrated benefits of periconceptional folic acid (FA) on neural tube defects (14), little is known about the impact on intellectual functioning in early childhood and beyond.

We have the unique opportunity to examine the effects of preconceptional micronutrient supplementation on offspring intellectual functioning using data that were collected prospectively from the offspring born to women who participated in a large randomized placebo-controlled trial (RCT) of preconception supplementation of multiple micronutrients (MMs) and iron and folic acid (IFA) compared with FA alone (15). We have previously shown that weekly supplementation with MMs or IFA improved linear growth and fine motor development at age 2 y compared with FA (16). In this article, we report findings on effects of preconception micronutrient supplementation on the full-scale IQ (FSIQ, primary outcome) and related subdomains (secondary outcomes) on the Wechsler Intelligence Scale for Children–Fourth Edition (WISC-IV) at 6–7 y. To our knowledge, this is the first study to report on the long-term effects of preconception micronutrient supplementation on child cognitive functions at school age.

## Methods

### Ethical approval

The study was approved by the Ethical Committee of Thai Nguyen National Hospital in Vietnam and Emory University's Institutional Review Board, Atlanta, Georgia, USA. The trial is registered at clinicaltrials.gov as NCT01665378. Written informed consent was obtained from all study participants.

### Study design, participants, and setting

Details of the parent preconception supplementation (PRECONCEPT) study have been published previously (15). In brief, we followed mother–child dyads who participated in the PRECONCEPT study, a double-blind RCT conducted in 20 communes located in 4 districts of Thai Nguyen province, Vietnam, from 2011 to 2013. A total of 5011 women of reproductive age were randomly assigned to receive weekly supplements containing 2800 μg FA, 60 mg iron and 2800 μg FA, or the same amount of IFA plus other micronutrients (**Supplemental Table 1**), from baseline until conception. During pregnancy, all women were switched to the standard of care and received daily IFA (60 mg iron and 400 μg FA) through delivery. All supplements were produced by NewCare (a Vietnamese pharmaceutical company) using premixes that were provided by DSM Nutritional Products and following the Good Manufacturing Practices standard. A total of 1813 women became pregnant, contributing to 1639 live births, of whom 1599 had known birth outcomes. The mean ± SD duration of preconceptional supplements was 33 ± 25 wk (range: 2–102 wk).

Follow-up visits were conducted in 2018–2019 when the offspring were 6–7 y of age. Commune health center staff and village health workers confirmed offspring survival, eligibility (we excluded offspring with congenital abnormality), and availability.

### Sample size and power calculation

Sample size calculations indicated that a final sample of 305 children per group would have at least 80% power to detect an effect size of 0.20 SD units or greater for the major outcomes at the end of the study, assuming a significance level of α = 0.05 for a 2-tailed test (17). We estimated that we would have an adequate sample size even after accounting for losses due to migration and deaths for the 1599 offspring in the PRECONCEPT cohort.

### Randomization and blinding

As described previously (15), both participants and study team were blinded to treatment assignment throughout the intervention and follow-up period. The 3 types of supplements were in capsule form; identical in appearance, smell, and taste; and coded with lot numbers at the factory that corresponded to the treatment arms.

### Outcome measures

The primary outcome of interest was child general intellectual functioning (FSIQ), which was assessed using the WISC-IV, an individually administered instrument for assessing the cognitive ability of children between the ages of 6 and 16 y (18). The WISC-IV consists of 10 core subtests: Vocabulary, Similarities, Comprehension, Block Design, Picture Concepts, Matrix Reasoning, Digit Span, Letter-Number Sequencing, Coding, and Symbol Search. This test has been validated and adapted in the Vietnamese context, including the translation, cultural analysis, modifications, and standardization (19). Specifically, the WISC-IV was first translated into Vietnamese by a bilingual psychologist fluent in Vietnamese and then back translated to English by an independent health researcher. All items were reviewed for cultural appropriateness by the Adaptation Committee and revised as needed. The modified version was piloted and standardized (19).

The WISC-IV was administered in a quiet room at an elementary school by well-trained researchers (pediatrician or researchers with master's degree in public health) who were trained by experts from the Vietnam National University, Hanoi for 2 wk. The training included classroom lectures and discussions, mock-interview field practice, and debriefing. All the test administrations during field practice were videotaped so that supervisors could score the test later to calculate interrater reliability; reliability estimates showed a high level of agreement (>0.8). The 12 researchers with the best performance were certified and participated in data collection. Each child was tested in a separate room to ensure no interruption. All children were provided with a snack before the testing and during the break time to make sure that they were not hungry when they took the test. The test took 70–90 min to administer. Researchers recorded all the answers for each item and each subset, and the psychometrician reviewed the scoring for each child after fieldwork was completed each day. Weekly field-based supervision was used for quality control of the assessment. Refresher training sessions were conducted every 6 mo after the initial training to ensure testing was conducted in a standardized manner.

The raw summary scores for each of the domains were computed using the relevant core subtests and then transformed to standardized composite scores (with mean ± SD of 100 ± 15; range: 40–160), using Vietnamese standardized norms to facilitate comparisons of the child's performance over time within and between individuals and across domains. The 4 specific cognitive domains include: *1*) Verbal Comprehension Index (VCI, a measure of the ability to understand, learn, and retain verbal information, as well as to use language to solve novel problems), *2*) Perceptual Reasoning Index (PRI, a measure of the ability to understand visual information and to solve novel abstract visual problems), *3*) Working Memory Index (WMI, a measure of the ability to hold verbal information in short-term memory and to manipulate the information), and *4*) Processing Speed Index (PSI, a measure of mental speed that may also be affected by factors such as attention). The FSIQ, the composite score that represents general intellectual ability, is a composite of the above domains (18, 20).

### Other potential covariates

We used data that were collected at the maternal, child, and household levels as potential covariates. These included measures of maternal nutritional status, namely, height, underweight [BMI (in kg/m^2^)  <18.5], anemia (hemoglobin <12g /L), mental health (high depressive symptoms defined as Center for Epidemiologic Studies Depression Scale score ≥10), and intellectual ability that was assessed using Raven's Progressive Matrices IQ Test (21) at the time of enrollment (preconception). Birth outcomes included birth size (weight, length), sex, preterm birth (<37 wk of gestation), low birth weight (<2500 g), and small for gestational age (SGA) that was defined as a birth weight below the 10th percentile for gestational age (22). Child dietary diversity was assessed as number of food groups consumed at 2 y and 6–7 y using the standard WHO guidelines (23), based on the maternal recall of all foods and liquids given to children in the last 24 h prior to the survey. Child enrollment in day care centers between the age of 0 and 36 mo or in preschools at 36–72 mo was used to assess early childhood learning environment.

We also evaluated the quality and quantity of the social, emotional, and cognitive support available to a child in the home environment using the Infant/Toddler Home Observation for Measurement of the Environment (HOME) Inventory and the Middle Childhood HOME Inventory at ages 1 and 6–7 y, respectively (24). These instruments were translated to Vietnamese and back translated by the first author and verified for construct validity during pretesting sessions. Results of the pilot test showed that the instrument was applicable to the Vietnam context but required minor modifications in the translated version to ensure linguistic and functional equivalence. Specifically, we adapted 5 items related to punishment to make it easier for the parents and field workers to report the occurrence of events (rather than the absence of events) and then reversed the coding during data analysis (25). The HOME was administered by trained personnel during home visits, and quality control was evaluated during weekly field visits by supervisors. The HOME scores for each group were rescaled to 0–100 to facilitate the comparison of home environment over time. Finally, household socioeconomic status (SES) index was constructed using a principal component extracted from multiple variables, including household ownership of different assets, livestock, house and land, and key housing characteristics (26), and then divided into tertiles.

### Statistical analysis

Data were first analyzed for all available samples with outcomes at 6–7 y, followed by the subgroup of children born to women who consumed the supplements for at least 26 wk before conception (per-protocol analysis) as we have done previously (27). We described selected baseline maternal and offspring characteristics by treatment groups and also compared baseline characteristics of study participants in the final analytic sample with those lost to follow-up.

For child intellectual functioning outcomes, we used ANOVA to test for overall differences in mean values among 3 treatment groups, followed by generalized linear regression analysis to estimate the differences in means for specific contrasts (MMs compared with FA and IFA compared with FA), adjusting for child age at follow-up and sex. Bonferroni corrections were used to correct the experiment‐wise error rate when using multiple tests. The adjusted estimates of the mean differences and 95% CIs of MMs compared with FA, as well as IFA compared with FA, were illustrated by the forest plot. We also conducted sensitivity analyses using a subsample that excluded twins (*n* = 20) and children who were born SGA, low birth weight, and/or preterm (*n* = 279) and controlled for assessors who administered the WISC-IV test.

We evaluated effect modification by testing interactions between treatment group with child sex, duration of supplementation (≥26 wk compared with <26 wk), baseline maternal BMI and anemia, tertiles of SES, and quality of home environment as we have done in previous analyses (16, 27). Because we found a significant interaction between treatment group with household SES, we also compared the mean differences (95% CIs) for specific contrasts, namely, MMs compared with FA and IFA compared with FA within in each tertile of SES. All reported *P* values were 2-tailed. Data were analyzed with STATA software version 16.0 (StataCorp).

## Results

### Study sample

**FIGURE 1:**
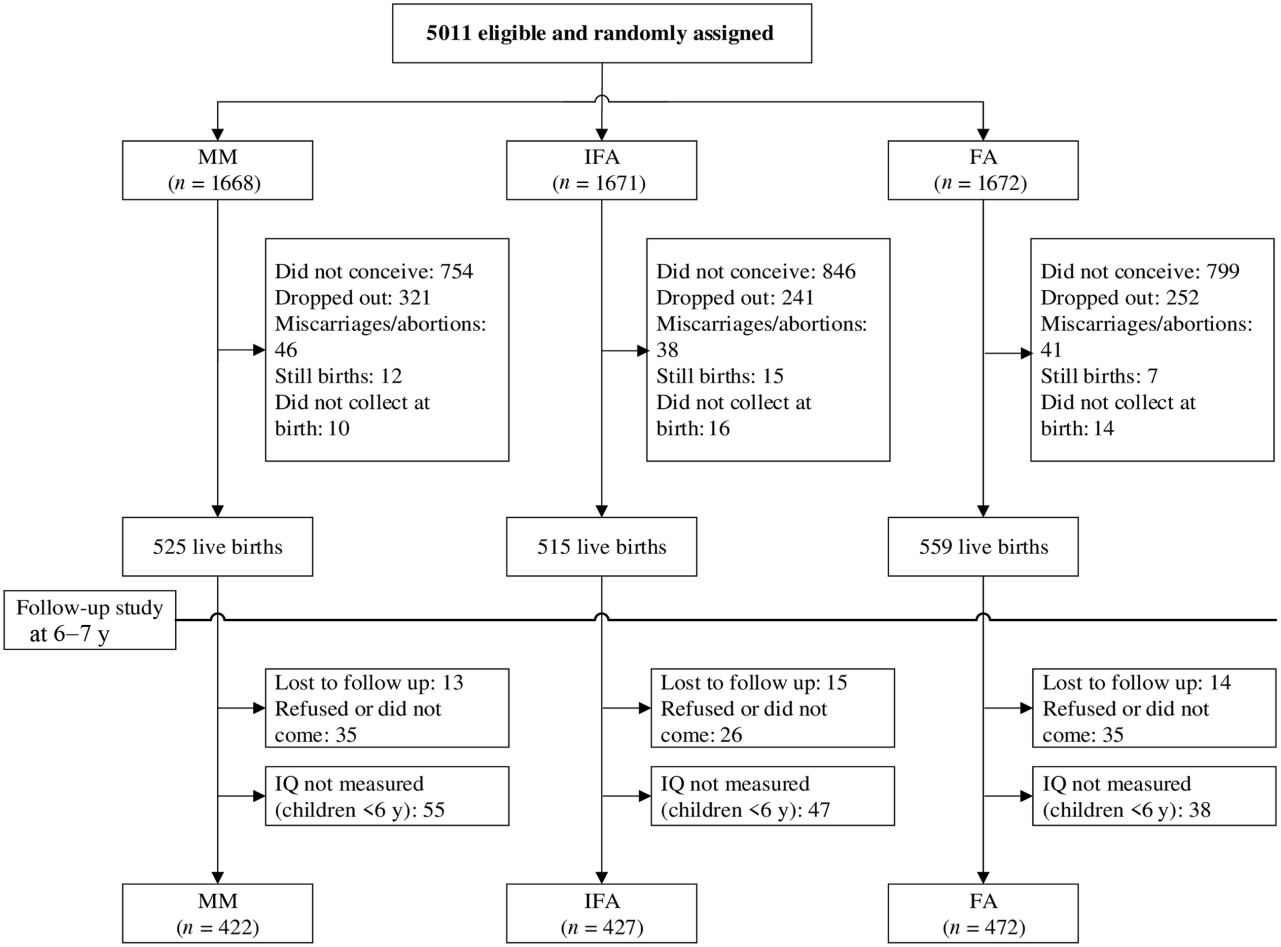
Flow diagram of participant progress throughout the study. FA, folic acid; IFA, iron and folic acid; MM, multiple micronutrient.

We successfully followed up 91% of the PRECONCEPT birth cohort (*n* = 1461 out of 1599 live births), of which 1321 children were aged ≥6 y and eligible for the WISC-IV assessment (**Figure 1**). The sample size for each group was at least 420, which gave us even more power to detect the expected effect size differences. We lost ∼3% (*n* = 42) of the cohort due to migration out of the study area (*n* = 29), dropout from the study (*n* = 5), or child death (*n* = 8). The proportion of children with missing data did not vary by treatment group. The final analytic sample was similar on most baseline characteristics to those with missing data (**Supplemental Table 2**).

There were no differences in baseline maternal and offspring characteristics at birth by treatment group for the analytic sample (**Table 1**). At the time of enrollment, women were on average aged 26 y, and >90% had 1 child. Approximately 30% of women were underweight and 20% were anemic. Mean offspring age at follow-up, quality of home environment, and dietary quality did not differ by treatment groups.

**TABLE 1:** Comparison of maternal preconception and child characteristics by treatment group^1^

Variable	MM (*n* = 422)	IFA (*n* = 427)	FA (*n* = 472)
Maternal characteristics at preconception enrollment			
Age, y	26.1 ± 4.7	25.9 ± 4.3	25.6 ± 4.3
Minority ethnic, %	53.8	48.2	49.8
At least high school education level, %	38.6	37.0	36.9
Work as farmers, %	82.0	78.9	79.4
Socioeconomic status index, *n*	0.0 ± 0.9	0.0 ± 0.9	0.0 ± 0.9
Number of children	0.96 ± 0.29	0.95 ± 0.31	0.95 ± 0.25
Number of children ≥1, %	94.2	93.2	94.3
Nutritional status			
Height, cm	153.0 ± 4.9	152.6 ± 4.9	152.9 ± 5.2
Weight, kg	46.2 ± 5.8	45.6 ± 5.1	45.9 ± 5.7
BMI, kg/m^2^	19.7 ± 2.0	19.6 ± 1.9	19.6 ± 2.1
Low BMI (<18.5), %	29.8	30.7	30.9
Hb, g/dL	12.9 ± 1.4	12.9 ± 1.4	13.0 ± 1.3
Anemia (Hb <12 g/dL), %	20.6	20.2	18.6
Poor maternal mental health, %	7.7	9.6	10.2
Maternal IQ	88 ± 16	88 ± 17	86 ± 17
Duration of supplementation, wk	28.5 ± 21.2	27.7 ± 20.7	28.7 ± 21.6
Compliance ≥80%, %	77.5	77.9	78.0
Received supplementation ≥26 wk, %	45.5	46.0	46.5
Child characteristics			
Female, %	50.4	50.1	44.4
Gestational birth, wk	39.1 ± 2.1	39.1 ± 1.9	39.2 ± 2.0
Preterm, %	11.2	9.3	9.3
Birth weight, g	3059 ± 407	3063 ± 422	3072 ± 422
Low birth weight, %	5.6	4.3	5.5
SGA, %	12.7	10.7	12.8
Enrolled in kindergarten during 0 to <36 mo, %	35.1	31.2	34.1
Attended 6–12 mo, %	23.0	19.7	22.9
Attended >1 y, %	10.9	10.5	10.0
Enrolled in preschool during 36–72 mo, %	99.8	100.0	100.0
Attended 6–12 mo, %	3.8	1.6	3.8
Attended >1 y, %	94.8	96.7	94.5
Current child age, mo	77.4 ± 4.0	77.7 ± 3.9	77.3 ± 3.9
Dietary diversity score at 2 y	5.0 ± 1.0	5.1 ± 1.0	5.0 ± 1.0
Dietary diversity score at 6–7 y	5.5 ± 1.5	5.5 ± 1.5	5.4 ± 1.6
Home environment			
Home environment at 12 mo	63.8 ± 7.9	63.3 ± 8.5	62.1 ± 8.5
Home environment at 6–7 y	56.3 ± 13.5	54.6 ± 14.7	55.1 ± 14.3

^1^Values are presented as mean ± SD unless otherwise indicated. High depressive symptoms defined as Center for Epidemiologic Studies Depression Scale ≥10. FA, folic acid; Hb, hemoglobin; IFA, iron and folic acid; MM, multiple micronutrient; SGA, small for gestational age.

### Micronutrient supplementation and child intellectual functioning

**FIGURE 2:**
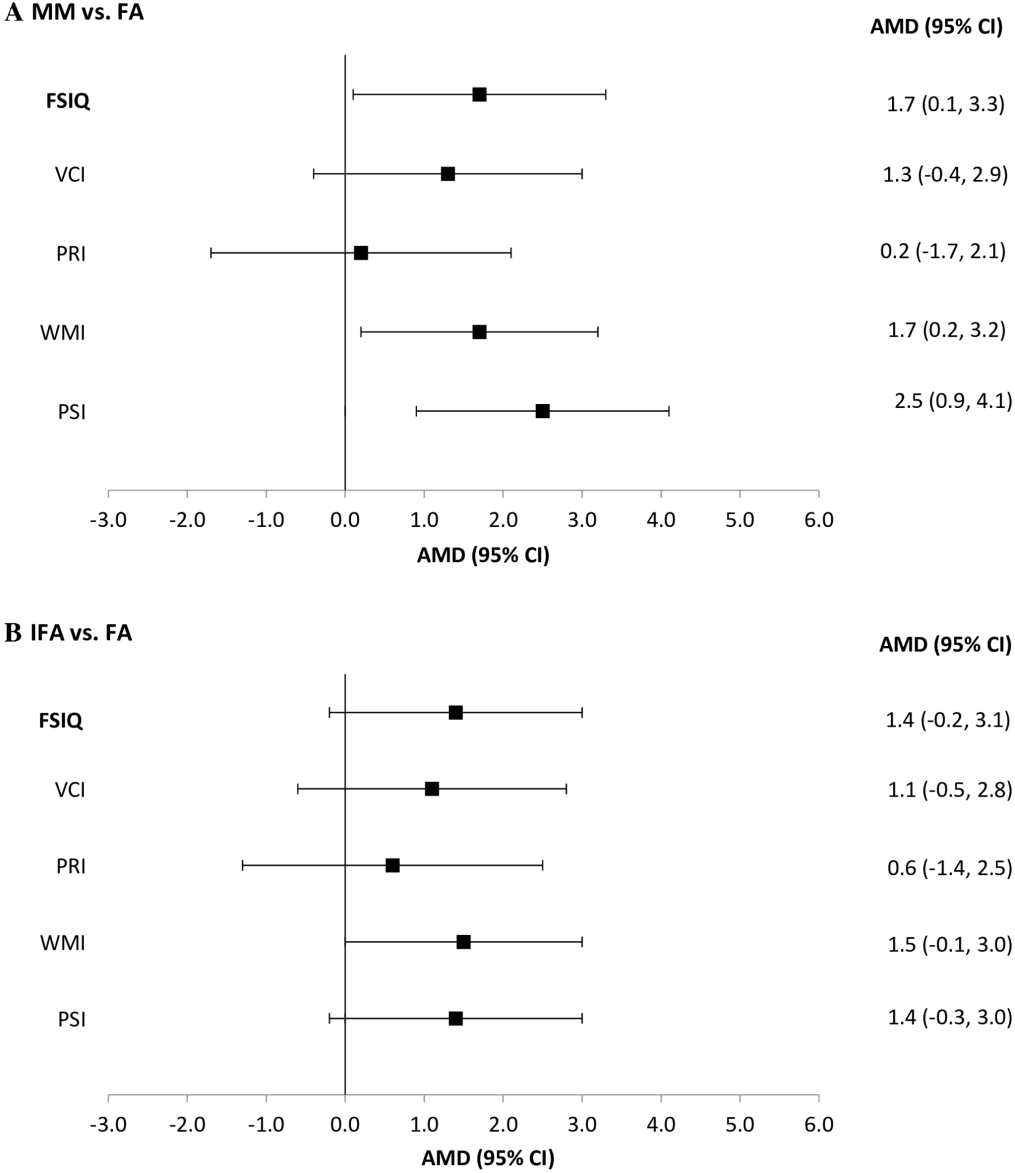
Differences in child intellectual functioning test scores by maternal supplementation group relative to folic acid group among children aged 6–7 y. MM compared with FA. IFA compared with FA. Generalized linear regression analysis to estimate the differences in means for specific contrasts (MM compared with FA and IFA compared with FA), adjusting for child age at follow-up and sex, *n* = 1289. AMD, adjusted mean difference; FA, folic acid; FSIQ, full-scale IQ; IFA, iron and folic acid; MM, multiple micronutrient; PRI, Perceptual Reasoning Index; PSI, Processing Speed Index; VCI, Verbal Comprehension Index; WMI, Working Memory Index.

Among children with available outcomes at ages 6–7 y (**Table 2**), mean FSIQ, WMI, and PSI differed by treatment group. Compared with the offspring in the FA group, those in the MM group had significantly higher mean FSIQ (β = 1.7; 95% CI: 0.1, 3.3), WMI (β = 1.7; 95% CI: 0.2, 3.2), and PSI (β = 2.5; 95% CI: 0.9, 4.1), after adjusting for age at measurement and sex (**Figure 2**). There were no significant differences by treatment group for VCI and PRI. These results were not altered in a sensitivity analysis that was done in a subsample that excluded multiple births (*n* = 20) and offspring who were born SGA, LBW, or preterm (*n* = 279) or when we controlled for assessors (data not shown).

**TABLE 2:** Comparison of child intellectual functioning scores by treatment group

Characteristic	MM, mean ± SD	IFA, mean ± SD	FA, mean ± SD	*P* value^1^
Overall analysis	(*n* = 422)	(*n* = 427)	(*n* = 472)	
Full-scale IQ	88.7 ± 11.9	88.9 ± 11.9	87.3 ± 12.7	0.095
Verbal Comprehension Index	82.1 ± 12.3	82.3 ± 12.5	81.1 ± 12.7	0.28
Perceptual Reasoning Index	93.0 ± 14.0	93.6 ± 14.6	92.9 ± 14.8	0.72
Working Memory Index	102.3 ± 11.3	102.3 ± 11.3	100.8 ± 12.1	0.067
Processing Speed Index	90.4 ± 12.5	89.5 ± 12.1	88.0 ± 12.3	0.012
Per-protocol analysis	(*n* = 184)	(*n* = 194)	(*n* = 216)	
Full-scale IQ	88.6 ± 11.5	87.9 ± 11.2	86.1 ± 13.0	0.085
Verbal Comprehension Index	82.6 ± 11.4	81.8 ± 11.7	80.1 ± 12.0	0.10
Perceptual Reasoning Index	92.7 ± 13.4	91.9 ± 14.3	91.8 ± 14.9	0.80
Working Memory Index	100.8 ± 11.2	102.5 ± 11.6	99.0 ± 12.5	0.010
Processing Speed Index	91.5 ± 12.4	89.1 ± 12.0	87.9 ± 13.0	0.016

FA, folic acid; IFA, iron and folic acid; MM, multiple micronutrient.

1 ANOVA test for comparison of means.

The effects of treatment were magnified in the subsample of offspring born to women who consumed supplements ≥26 wk before conception (**Supplemental Figure 1**). Compared with the offspring in the FA group, those in the MM group had significantly higher FSIQ (β = 2.4; 95% CI: 0.1, 4.8) as well as VCI (β = 2.4; 95% CI: 0.1, 4.7) and PSI (β = 3.4; 95% CI: 1.0, 5.9). Offspring in the IFA group also had significantly higher WMI (β = 3.4; 95% CI: 1.1, 5.7) compared with the FA group. There were no statistically significant differences by treatment group for PRI.

**FIGURE 3:**
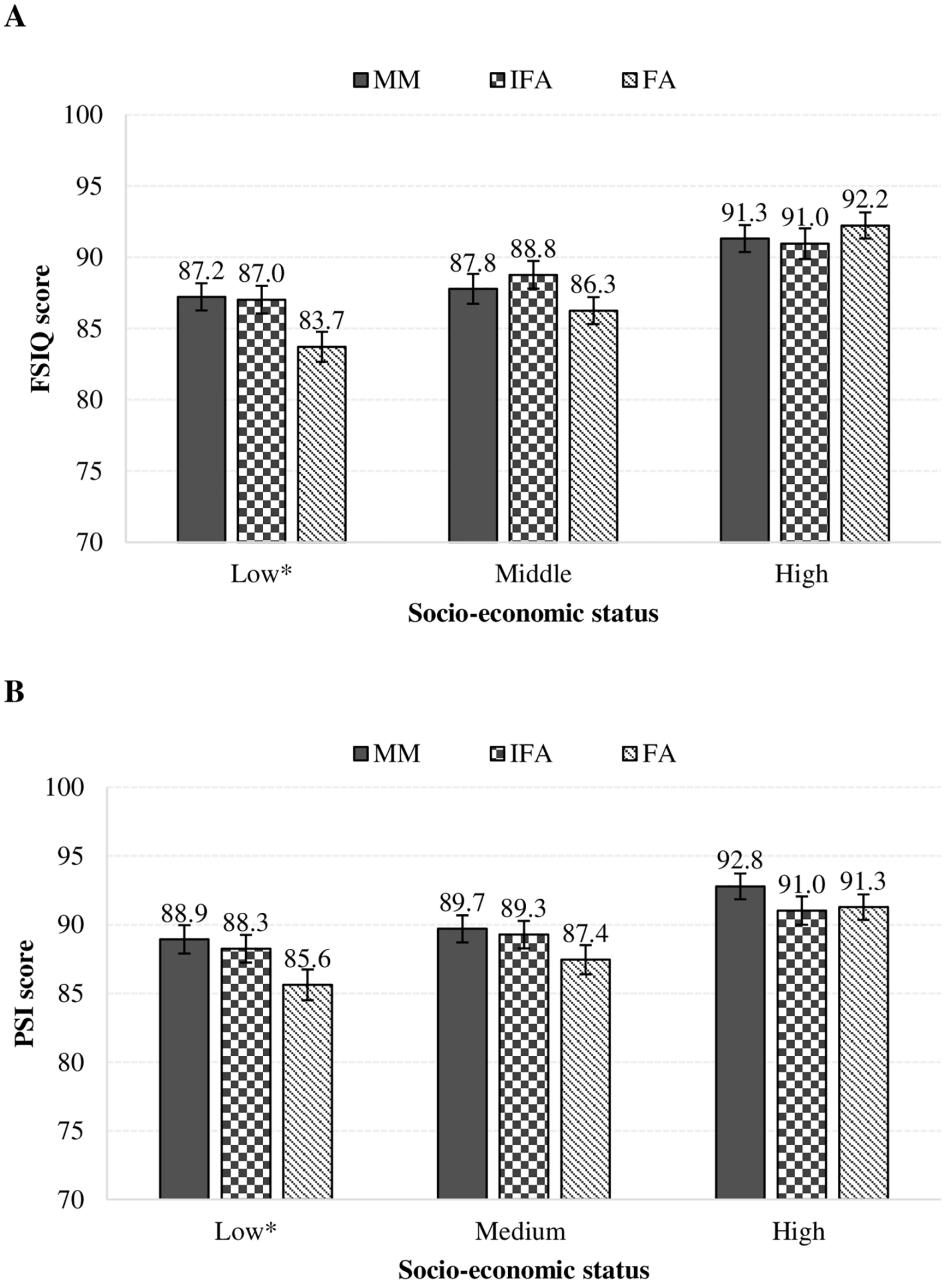
Relation between socioeconomic status (SES) and Processing Speed Index (PSI) (A) and full-scale IQ (FSIQ) (B) at 6–7 y of age by intervention group. Effect modification was evaluated by testing interactions between treatment group with household SES tertile. For PSI, *P* for overall interaction = 0.04; for FSIQ, *P* for overall interaction = 0.08. Values are mean and SE, *P < 0.05. FA, folic acid; IFA, iron and folic acid; MM, multiple micronutrient.

Finally, we found evidence of an interaction between treatment group and tertile of household SES at baseline for FSIQ (*P* = 0.08) and PSI (*P* = 0.04) (**Figure 3**). In the subgroup of participants belonging to the bottom tertile of SES, offspring born to women receiving preconception MMs and IFA had higher FSIQ (β = 3.5; 95% CI: 0.8, 6.2 for MMs and 3.3; 95% CI: 0.6, 6.0 for IFA) and PSI (β = 3.3; 95% CI: 0.6, 6.0 for MMs and 2.6; 95% CI: –0.06, 5.3 for IFA) compared with those born to women receiving only FA. There were no differences by treatment group for those in the middle and top tertiles of SES. We did not find evidence of an interaction between treatment group and sex, maternal anemia or underweight at baseline, or HOME scores at baseline or 6–7 y of age.

## Discussion

Offspring born to women who received MM supplement prior to conception had higher FSIQ, WMI, and PSI compared with those born to women who received only FA. Moreover, this effect was greatest among children born in families with the lowest SES. The impact of preconception multiple micronutrient supplementation on child intellectual functioning was also magnified among children whose mothers consumed supplements at least 6 mo before conception.

Our findings of improved outcomes at ages 6–7 y build on our previous findings of effects of the intervention on measures of fine motor development at age 2 y (16) and may reflect the importance of more robust, comprehensive measurement of intellectual functioning at later ages that includes measures of component abilities such as information processing speed and memory (28). Our findings are similar to other studies that have evaluated the long-term effects of prenatal nutrition interventions (29). For example, the INCAP (Institute of Nutrition of Central America and Panama) longitudinal study in Guatemala observed the cognitive benefits of prenatal supplementation with a nutrition supplement only after the children started school (30), and these effects increased with age (31–33). Similarly, a study in Mexico found that prenatal supplementation with DHA had an impact on child development among children with lower home environment scores that became apparent at 18 mo of age (34) and persisted through age 5 y (35). A follow-up of the SUMMIT (Supplementation with Multiple Micronutrients Intervention Trial) study in Indonesia also found that prenatal MM supplementation had long-term benefits for child cognitive development at ages 9–12 y (36). Finally, prenatal MM supplementation was associated with increased adolescent intellectual development among children aged 10–14 y in a trial from China (37).

There has been increasing emphasis on intervening before pregnancy because maternal nutritional status during the preconception period might determine lifelong health and achievement (38). Evidence from animal and observational studies indicates that the nutritional and health status of women as they enter pregnancy may play a key role in placental function and subsequent growth and development of the fetus later in life (7, 39). Periconceptional nutrition may also influence offspring health and cognitive outcomes by affecting the growth and development of key organs, such as the brain, liver, and pancreas, during the first few weeks of pregnancy (40). To date, only a few RCTs (41–43) have evaluated the effects of a preconception nutrition intervention trial beyond neural tube defects (38), and none have evaluated the benefits for intellectual functioning. For example, the Mumbai Maternal Nutrition project found higher offspring birth weight among women who received a micronutrient-rich snack daily before conception and throughout pregnancy compared with the control group (41), and the Women First trial, a multicountry RCT, also found increased birth length and reductions in newborn stunting among women who received a daily lipid-based micronutrient supplement that began ≥3 mo prior to conception (42, 43). The present study expands on these findings to demonstrate a long-term impact of preconception supplementation on child intellectual functioning. Micronutrient availability, especially during the first trimester of pregnancy through early infancy, may influence the development of the brain's macro-structure (e.g., the hippocampus) and micro-structure (e.g., myelination of neurons), which are involved in processes related to attention, memory, intelligence, and sensory perception (44, 45). These biological mechanisms may explain the improvements in PSI (a measure of mental speed that may be affected by attention) and WMI (the ability to hold verbal information in short-term memory and to manipulate the information) that we observed. In contrast, the lack of improvement in higher-level functions that are related to language and abstract reasoning based on visual function needs to be explored further. It is possible differences may emerge later and/or these domains are more sensitive to the adequacy of dietary intakes and the quality of the learning environment after the first 1000 d (46).

Meta-analyses have shown the weighted mean effect size on child development of 0.04 SD for micronutrient interventions during the prenatal period, 0.08 SD for interventions in the postnatal period (47), and 0.14 SD for interventions in school-age children. This result is consistent with the effect sizes reported in our study of 0.12–0.15 SD on different cognition domains from preconception interventions. Although we could have expected to see larger differences among those who received MMs compared with IFA based on the roles of different micronutrients on brain development (5, 6), this was not the case in our study; the effect size of these 2 treatments compared with FA alone was similar.

Finally, our results also indicate that preconception micronutrient supplementation may decrease socioeconomic disparities in both overall intellectual functioning and processing speed index. Consistent with previous work (4), we found positive associations between household SES and measures of intellectual functioning, but these associations were significantly attenuated among offspring born to women who received preconception MMs and IFA compared with FA only. Poverty is associated with developmental delays before 12 mo, with increasing deficits later on through different pathways such as nutritional deficiencies, poor living condition, and less-stimulating home environments (48). It is possible that preconception MM or IFA supplementation compensated for some of these adverse effects.

To our knowledge, this study is the first to evaluate the impact of preconception micronutrient supplementation on child intellectual functioning at 6–7 y of age. Our study has several strengths, including the double-blinded RCT, the large sample size, high supplement compliance (49), and relatively low loss to follow up (∼17%). The development outcomes were measured using the WISC-IV, which is well adapted and standardized in the Vietnamese context with high validity and reliability (19).

Potential limitations of the study include the lack of data on the quality of the early childhood learning environment in daycare settings and/or preschool. However, since the treatment groups remained balanced on several baseline characteristics, we expect the same for the unobserved characteristics. Finally, since we provided FA to the control group for ethical reasons, we may have underestimated the benefit as we do not have a true control group.

In conclusion, our findings provide novel evidence on the benefits of providing multiple micronutrients before conception for offspring development and learning during the preschool years. These results support the importance of expanding current efforts that focus on the first 1000 d by investing in women's health and nutrition even before they get pregnant. Our findings on offspring cognition, combined with findings from other preconception studies for improved birth outcomes (41–43), provide valuable evidence of both clinical and public health relevance. Specifically, ensuring optimal health and nutritional status of women of childbearing age before they get pregnant should receive high priority in clinical practice that typically focuses only on the provision of antenatal care. This could be done by combining with the provision of reproductive health services at various life stages, beginning in adolescence through postpartum. Our findings also have important implications for policymakers and public health programs that include interventions to improve dietary intakes and promote micronutrient supplementation beyond FA for women of reproductive age. Finally, our findings support the importance of conducting long-term follow-up studies to demonstrate benefits on the various aspects of brain function and learning that are critical for human capital formation (50).

## Supplementary Material

nqaa423_Supplemental_File

## Data Availability

Data described in the manuscript, code book, and analytic code will be made available upon request.

## Notes

Funding for this research was provided by the Nestle Foundation, the Mathile Institute for the Advancement of Human Nutrition, and the Micronutrient Initiative. The funders had no role in study design, data collection and analysis, data interpretation, decision to publish, or preparation of the manuscript.

Supplemental Tables 1 and 2 and Supplemental Figure 1 are available from the “Supplementary data” link in the online posting of the article and from the same link in the online table of contents at https://academic.oup.com/ajcn/.

Abbreviations used: FA, folic acid; FSIQ, full-scale IQ; HOME, Home Observation for Measurement of the Environment; IFA, iron and folic acid; MM, multiple micronutrient; PRECONCEPT, preconception supplementation; PRI, Perceptual Reasoning Index; PSI, Processing Speed Index; RCT, randomized controlled trial; SES, socioeconomic status; SGA, small for gestational age; VCI, Verbal Comprehension Index; WISC-IV, Wechsler Intelligence Scale for Children–Fourth Edition; WMI, Working Memory Index.
