# Supplementary material for: Preconception micronutrient supplementation positively affects child intellectual functioning at 6 y of age: A randomized controlled trial in Vietnam
Source: Am J Clin Nutr. 2021 Mar 1;113(5):1199–208. doi: 10.1093/ajcn/nqaa423 (PMC8106753; doi:10.1093/ajcn/nqaa423)
Supplement: nqaa423_Supplemental_File [file nqaa423_supplemental_file.docx]

**Supplemental Table 1: The composition of preconception micronutrient supplements**

| **Ingredient** | **Pre-pregnancy (weekly)** | | | **RDA for non-pregnant women^1^** |
| --- | --- | --- | --- | --- |
|  | **MM** | **IFA** | **FA** |  |
| Vitamin A, μg | 800 |  |  | 700 |
| Vitamin D, IU | 600 |  |  | 15 |
| Vitamin E, mg | 10 |  |  | 15 |
| Vitamin C, mg | 70 |  |  | 75 |
| Thiamine, mg | 1.4 |  |  | 1.1 |
| Riboflavin, mg | 1.4 |  |  | 1.1 |
| Niacin, mg | 18 |  |  | 14 |
| Vitamin B_6,_ mg | 1.9 |  |  | 1.3 |
| Vitamin B_12,_ μg | 2.6 |  |  | 2.4 |
| Folic acid, μg | 2800 | 2800 | 2800 | 400 |
| Iron (ferrous sulfate), mg | 60 | 60 |  | 18 |
| Zinc (sulfate), mg | 15 |  |  | 8 |
| Copper, mg | 2 |  |  | 0.9 |
| Selenium, μg | 65 |  |  | 55 |
| Iodine, μg | 150 |  |  | 150 |

^1^IOM. Dietary Reference Intakes (DRIs): Recommended Intakes for Individuals. Food and Nutrition Board, Institute of Medicine, National Academies, 2004. FA: Folic Acid, IFA: Iron and Folic Acid, MM: Multiple Micronutrient, RDA- Recommended Daily Allowance

**Supplemental Table 2: Comparison of baseline characteristics of participants in the final analytic sample and those missing data at follow-up^1^**

| **Variable** | **Final sample**  ***(n= 1321)*** | **Missing data at age-6-7 y**  ***(n= 278)*** |
| --- | --- | --- |
| **Maternal characteristics at preconception enrollment** |  |  |
| Age, *y* | 25.9 ± 4.4 | 26.3 ± 3.9 |
| Minority ethnic, *%* | 50.6 | 39.3 |
| At least high school education level, *%* | 37.5 | 37.1 |
| Work as farmers, *%* | 80.1 | 81.4 |
| Socio-economic status index | 0.0 ± 0.9 | 0.0 ± 1.0 |
| Number of children ≥ 1, *%* | 93.9 | 97.7 |
| Nutritional status |  |  |
| Height, *cm* | 152.8 ± 5.0 | 152.0 ± 5.5 |
| Weights, *kg* | 45.9 ± 5.5 | 45.6 ± 6.0 |
| BMI, *kg/m^2^* | 19.6 ± 2.0 | 19.7 ± 2.3 |
| Low BMI (BMI <18.5), *%* | 30.5 | 33.1 |
| Hb, *g/dl* | 12.9 ± 1.3 | 12.9 ± 1.4 |
| Anemia (Hb<12 g/dL), *%* | 19.8 | 21.4 |
| **Child characteristics** |  |  |
| Female, *%* | 48.2 | 60.4 |
| Gestational birth, *wk* | 39.2 ± 2.0 | 39.5 ± 1.7 |
| Preterm, *%* | 9.9 | 5.0 |
| Birth weight, *g* | 3065 ± 417.0 | 3234 ± 652.0 |
| Low birth weight, *%* | 5.1 | 5.9 |
| SGA, *%* | 12.1 | 12.5 |
| Current child age, *mo* | 77.4 ± 3.9 | 68.8 ± 2.9 |
| Dietary diversity score at 6-7y | 5.5 ± 1.5 | 5.7 ± 1.4 |
| **Home environment** |  |  |
| Home environment at 12 months | 28.4 ± 3.7 | 29.7 ± 3.3 |
| Home environment at 6-7y | 10.0 ± 2.6 | 9.7 ± 2.5 |

^1^t-test for comparison of means and chi-square test for comparison of proportions, all p-values >0.05 for comparisons by intervention group. Hb: hemoglobin; SGA: small for gestational age.

**Supplemental Figure 1: Differences in child development test scores by maternal supplementation group relative to folic acid group among children aged 6-7y, results from per protocol analysis^1^**

^1^Generalized linear regression analysis to estimate the differences in means for specific contrasts (MM vs. FA and IFA vs. FA), adjusting for child age at follow-up and sex; AMD: adjusted mean difference, CI: confidence interval, FA- Folic Acid, IFA- Iron and Folic Acid, MM- Multiple Micronutrient, IQ: Intelligence quotient, PRI: Perceptual reasoning index, PSI: Processing speed index, VCI: Verbal comprehension index, WMI: Working memory index, FSIQ: Full scale IQ.
